# P05.31. Development of a manualized protocol of massage therapy for clinical trials in osteoarthritis

**DOI:** 10.1186/1472-6882-12-S1-P391

**Published:** 2012-06-12

**Authors:** A Ali, J Kahn, L Rosenberger, A Perlman

**Affiliations:** 1 Yale University School of Medicine, Derby, USA; 2 University of Vermont, Burlington, USA; 3 Yale-Griffin Prevention Research Center, Derby, USA; 4 Duke University, Durham, USA

## Purpose

Manualization is considered to be an integral methodologic component for rigorous research on complementary and alternative therapies. Clinical trial design of manual therapies may be especially challenging since techniques are often individualized and practitioner-dependent.

## Methods

Here we describe our methodology in creating a standardized and reproducible massage intervention tailored to subjects with osteoarthritis of the knee while respectful of the individualized nature of massage therapy.

## Results

The manualized protocol specifies the body regions to be addressed, with distinct 30 and 60-minute protocols, as well as the standard Swedish strokes to be used (effleurage, petrissage, tapotement, vibration, friction, and skin rolling).

## Conclusion

The resulting massage protocol was made reproducible, using standard massage techniques, as well as being flexible for individual subject variability. Aside from being reproducible this manualized Swedish massage protocol has successfully been used in a dual-site dose-finding clinical trial.

